# On the Theory and Practice of Midwifery

**Published:** 1846-03

**Authors:** 


ART. VII.—*On the Theory and Practice of Midwifery.* By FLEETWOOD CHURCHILL, M. D., M. R. J. A., Licentiate of the College of Physicians in Ireland; Physician to the Western Lying-in Hospital; Lecturer on Midwifery, &c., in the Richmond Hospital School of Medicine; Author of "a Treatise on the Diseases of Females," &c. &c. *With Notes and Additions*, by ROBERT M. HUSTON, M. D., Professor of Materia Medica and General Therapeutics, and formerly of Obstetrics and the Diseases of Women and Children, in the Jefferson Medical College of Philadelphia; President of the Philadelphia Medical Society, &c. &c. Second American edition. With one hundred and twenty-eight illustrations from drawings by Bagg and others. Engraved by Gilbert. Philadelphia: Lea & Blanchard. 1846. (8vo. pp, 525.)

From the title-page the reader will be able to divine the character of this work, even if he should never have heard of it before; which, however, is by no means a probable case. The author of it is one of the masters in the obstetric art, whose name is known to every reader of English medical literature. His work on the "Diseases of Females," as well as the volume, the second edition of which stands at the head of this article, has been widely circulated in our country, and much esteemed by the profession everywhere. The form in which his "Midwifery" now appears, will recommend it equally to the student, seeking to learn the first principles of the science, and the practitioner, desirous of knowing the opinions of an experienced and judicious writer on any point of practice. Without being as learned as Ramsbotham, its references are numerous enough for the purposes of most readers, and its practical directions are given with clearness, and at sufficient length. Its illustrations are admirable, and with its full index, clear type, fair paper, and substantial binding, it forms a volume worthy of a place among the standard works on Obstetric Medicine.

We have just risen from the perusal of Dr. Churchill's chapter on *Phlegmasia dolens*, a case of which under our charge has caused us to consult all the authorities relative to that affection within our reach. Of course, in looking through many treatises on the same subject, we have met with much repitition, especially in the works recently published; but in none did we find a fuller or more satisfactory account of all that pertains to the disease than is given by the volume before us.

It will be singular indeed, if, with such writers as our times have produced, and a press teeming with works in which art strives successfully to exhibit nature as she is, we should not have an accomplished race of obstetricians. The study of books, in which all is made as intelligible as the mechanism of labor is rendered by the illustrations of this volume, is not a toil, but a pleasure.

The rank which women hold in society is deemed to be a pretty sure criterion of the civilization of a people. Among savages they are slaves, but as men rise above a state of barbarism, they grow respectful towards the weaker sex, indulgent to their weaknesses, and studious of their happiness. If our profession be tried by this test, it must be pronounced eminently civilized. No branch of the healing art is cultivated with more zeal or assiduity than obstetrics; hardly any other has called forth, in latter years, so many excellent productions.

